# Supplementary material for: The Fidelity of Rheumatoid Arthritis Multivariate Diagnostic Biomarkers Using Discriminant Analysis and Binary Logistic Regression
Source: Biomolecules. 2023 Aug 25;13(9):1305. doi: 10.3390/biom13091305 (PMC10526504; doi:10.3390/biom13091305)
Supplement: Supplementary file 1 [file biomolecules-13-01305-s001.zip › biomolecules-2535160-supplementary-conversion/biomolecules-2535160-supplementary.docx]

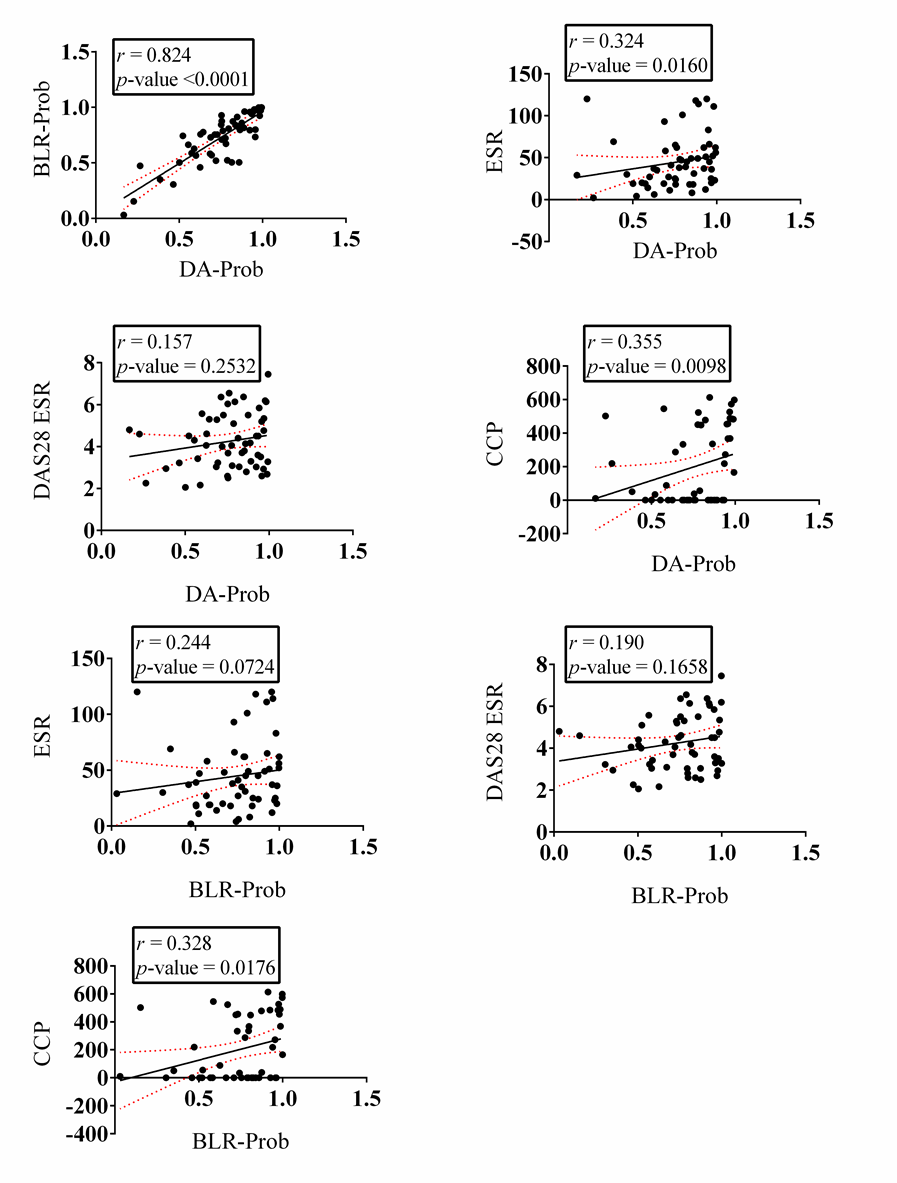


**Figure S1.** Estimated probability of RA diagnosis correlates with erythrocyte sedimentation rate (ESR), disease activity score-28 with erythrocyte sedimentation rate (DAS28ESR), and cyclic citrullinated peptide antibody (Anti-CCP). Analysis done on female RA patients with available clinical data (n = 55). DA-Prob: probability estimated using discriminant analysis, BLR-Prob: probability estimated using binary logistic regression.
